# Mr. Davies, on the Effects of Cerussa Acetata

**Published:** 1808-01

**Authors:** Robert Davies

**Affiliations:** Member of the Royal College of Surgeons, London; and Extraordinary Member, Roy. Med. Soc. Edin.


### *To the Editors of the Medical and Physical Journal.*

GENTLEMEN,

*Piddletown, Dorset, Nov.* 13, 1807.

YOU will oblige me by inserting the following cases, illustrative of the effects of Cerussa Acetata administered internally, in your Publication.

I am, &c.

ROBERT DAVIES,

Member of the Royal College of Surgeons, London; and Extraordinary Member, Roy. Med. Soc. Edin.

---

*Oct.* 18, 1806. I was called to see W. Toms, a man advanced in years, labouring under an hæmorrhagy from the lungs. He was affected in the same way before, without being able to attribute it to any particular cause. In the present instance, the disease appears to have been brought on by the the violent action of a strong emetic, administered by an empiric in the neighbourhood. When I saw him, he had thrown up a considerable quantity of frothy florid blood; his pulse was quick and irregular, and he appeared in danger of suffocation.—℞. Cerussæ acetat. ℈j. Conserv. rosæ ℥i. tere, et divide in bol. No. vj. sum. unum 2a. quâque horâ.—He had not any considerable discharge of blood from the time of taking the first bolus, but continued spitting clots for several days after. After the cerussa acetata, I ordered the acid. vitriol. cum infus. rosæ, with the occasional use of opening medicines. His restoration to health was gradual, nor has he had any return of the complaint.

*Nov.* 22, 1806.—Mrs. S—— has had several children born alive; but, in the five last periods of pregnancy, she aborted about the fifth month; except in one instance, when she went the full time of gestation, and was delivered of a child which appeared to have been dead some time. She was, at the above date, in the fourth month of pregnancy, and affected with uterine hæmorrhagy to a considerable degree.—℞. Cerussæ acetat. ℈j. Micæ panis et mucilaginis gum. arab. q. s. fiat massa in pil. No. xx. divid. sum. unam 4a. q. horâ. cum gutt. x. tinct. opii ex aquâ.

23. The hæmorrhagy has ceased; the quickness of pulse, and feverish symptoms, abated. I directed her to continue the use of her medicines as before.

24. Yesterday evening the discharge returned, when she applied vinegar and water externally; it ceased in the course of the night. She continued the medicine for two days after; she went out her full time, and was delivered of a fine healthy infant.

*April* 16, 1807.—I saw —— Giffard, a young woman of plethoric habit, labouring under symptoms of pneumonia. I thought it a fair case for trying the effects of cerussa acetat. administered internally in inflammatory cases. However, as the symptoms ran high, and her situation appeared critical, I was afraid to omit the obvious means of giving her relief, and accordingly took off about fourteen ounces of blood. Half an hour after the blood-letting, her pulse was reduced from 98 to 86 pulsations in a minute. Sumat ceruss. acetat. gr. iij. ex melle. In two hours after, her pulse was reduced to 80; her belly was costive. Sumat pulv. antimon. gr. iij. calomelanos gr. vj.

17. I

17. I found her symptoms aggravated; the quickness and fulness of her pulse increased; her skin hot and dry. The medicine procured her two dark-coloured offensive stools. Mittatur sanguis ad ℥vij. sum. cerussæ acetatæ gr. ij. ex melle 2a. quâque horâ per diem, et horâ somni repetat. pulverem ex calomelane et pulv. antimon.

18. Spent a better night than heretofore; has had two motions more natural in their appearance; her pulse still continues full and quick; she expectorates freely. Cont. ceruss. acetat. et sum. tinct. op. camph. gutt. xx. ex aquâ quartâ quâque hora.

19. Feels much better, and complains of nothing but weakness.

22. Was called to see her again, and found her labouring under distinct symptoms of acute hepatitis. The hardness of her pulse induced me to use the lancet again. I took off ten ounces; a blister was directed to her side.—℞. Calomelanos, pulv. antimon. aa gr. iij. fiat pulv. quartâ quâque horâ sumend.

23. Pain easier, cough troublesome, a good deal purged by the medicine. Sum. tinct. opii, camph. gutt. xxx. quartâ quâque horâ. Continuatur pulv. cal. et ant.

24. Pain gone, cough easier, purging continues; breath and gums affected by mercurial action. Omit. pulv. antimon. et calomel; sumat. tinc. opii gutt. x. tertiâ quâque horâ donec diarrhea cessaverit. From this period she gradually recovered.

No *decided* inference in favour of the exhibition of cerussa acetata can be drawn from this case; the dangerous state of my patient, from the commencement, prevented my venturing to give it a fair trial. It certainly, however, appeared to have lessened the inflammatory symptoms on the days she took it. As her complaints appeared, *a priori*, connected with a diseased liver, *her cure* may be attributed to the effects of calomel, in conjunction with bloodletting.

*July* 17, 1807.—Betty Luccas, of a weakly constitution and spare habit, in the fourth month of pregnancy, and affected with uterine hæmorrhagy.—Mittatur sanguis ad ℥viij.—℞. Infus. rosæ ℔ß. acid vitriol. dilut. gutt. xxx. tinct. opii gutt. xl. M. sum. ℥iß. 3a. q. h.—℞. Kali vitriolat. pulv. rhei aa. gr. x. omni nocte sumend.

20. She aborted; retained the placenta; the discharge not not very profuse. I ordered her a saline mixture, with tinct. opii.

22. The placenta came away entire.

24. She appeared very low and weak, her pulse fast. She attributed her complaints to the profuseness of the lochial discharge. Sum. decoct. cinch. acidulat. ℥ij. cum tinct. op. gutt. x. quater de die.

The discharge continued, without alteration, till the 30th; when the bark disagreeing with her stomach, I ordered the following.—℞. Gum. kino. catechu, extract. hematox. alum. commun. aa. ℈i. mucilaginis q. s. fiat mass. in pil. xxiv. divid. sum. iij. quartâ quâque horâ.

The discharge continued, though in less quantity, till August 4, when I was sent for in a hurry to see her. About four hours before I came, she was seized with violent pain and flooding, which continued *in a very alarming degree*; her pulse was fast and *undulating*; the quantity she had discharged was almost incredible, and the hæmorrhagy continued in a stream.—℞. Ceruss. acetat. ℈i. conserv. rosæ ℥i. tere et divide in bol. vj. sum. unum statim.

I directed cloths, wrung out of vinegar, to be firmly applied to the parts; cold air to be admitted to the room, and attention paid to her posture, &c. &c. A little after she took the bolus, the change in her pulse was remarkable; it became slow and equable. When the cloths were changed, the flooding appeared to have ceased.—Rep. bolus 2a. quâque horâ.

She has not had any return of the complaint, and has gradually recovered her strength.

I have exhibited cerussa acetata in two cases of chronic menorrhagia, in women advanced in years; in one it probably increased the disease; but in neither instance was it productive of any advantage. Both yielded to the decoct. cinchonæ, with increased doses of opium.

No unpleasant symptoms occurred in any of the above cases, that could be attributed to the deleterious action of lead; except a tendency to costiveness, which was easily obviated. Its effects, on lowering the pulse, were very perceptible.—From these circumstances, I am led to believe its modus operandi is, by diminishing nervous irritability; and, consequently, lowering the circulation, it appears a *direct sedative*, and promises to be of use, exhibited internally in all cases of internal inflammation; and in those instances where digitalis has been recommended, with with a view of lessening the action of the arterial system. It may, also, be found efficacious in preventing habitual abortion; which complaint is, at present, justly ranked among the *opprobria medicorum.*

The internal exhibition of cerussa acetata, is much decried by some practitioners, and condemned as altogether unsafe. An opinion, undoubtedly, drawn from the supposed action of lead, in producing that dreadful complaint, Colica Pictonum.

Prejudice is carried so far against this medicine, as, in many cases, to prevent its external use; through fear of unpleasant effects being produced by its absorption. In military practice, no plan that has hitherto been pursued, so speedily, certainly, and effectually, heals the backs of punished men, as the application of cloths, wet constantly with a solution of cerussa acetata.

During a few years practice in the army, I have had the care of men who received very severe punishments; but by persisting in the use of cerussa acetata, I never had a case attended with one of the *many* unpleasant symptoms which so frequently occur.

Still several military medical men, who were witnesses to the success of that practice, sooner than risk absorption from so large a wounded surface, preferred having recourse to the whole farrago of suppurants, digestives, &c. The surgeon of the first regiment I served in, assured me, that he had followed this mode of treatment for many years; in tropical climates, in hospitals, where contagion prevailed, and with other disadvantages, with invariable success. Nor has he ever seen any constitutional effects, which could be possibly attributed to the absorption of lead.

Mr. Edwards, surgeon in Dorchester, mentioned two instances to me, in which a drachm of cerussa acetata had been taken by mistake, without producing any very unpleasant symptom. In one case, a pain in the bowels, which was removed by a dose of castor oil; in the other, a constipated state of the bowels, for three or four days, which likewise yielded to an opening medicine.

Without presuming to controvert what, I believe, is admitted as a medical fact—that lead, whether taken in a state of sublimation into the lungs, or by the stomach, or some other means, into the system, is the cause of Devonshire colic; still it appears to me, that the preparation we are speaking of, at least in moderate doses, is exempt from those deleterious qualities. We know that various comcombinations of a medicine, will produce various effects; we need only instance those of mercury.

Dr. Duncan, of Edinburgh, in his very valuable work, the Edinburgh New Dispensatory, in the article Acetis plumbi, says, "The internal use of acetate of lead, notwithstanding the encomiums some have been rash enough to bestow upon it, is entirely to be rejected." This, from such high authority, makes a tyro in the profession tremble, when he asserts a contrary opinion. However, I conceive it the duty of every professional man, to state the result of his practice, when there is a chance of promoting the advancement of science: I consequently with diffidence commit the foregoing cases to the perusal of such as will take the trouble of reading an article from the pen of a man unknown in the medical world, and hope that some one, whose professional celebrity will sanction the exhibition of a medicine, that promises to be of extensive use, will give it a fair trial, and make public the result of his practice.

